# Detection of tyrosine kinase inhibitors-induced COX-2 expression in bladder cancer by fluorocoxib A

**DOI:** 10.18632/oncotarget.27125

**Published:** 2019-08-27

**Authors:** Jennifer Bourn, Sony Pandey, Jashim Uddin, Lawrence Marnett, Maria Cekanova

**Affiliations:** ^1^ Department of Small Animal Clinical Sciences, College of Veterinary Medicine, The University of Tennessee, Knoxville, TN 37996, USA; ^2^ University of Tennessee and Oak Ridge National Laboratory, Graduate School of Genome Science and Technology, The University of Tennessee, Knoxville, TN 37996, USA; ^3^ A. B. Hancock, Jr., Memorial Laboratory for Cancer Research, Departments of Biochemistry, Chemistry and Pharmacology, Vanderbilt Institute of Chemical Biology, Center for Molecular Toxicology and Vanderbilt-Ingram Cancer Center, Vanderbilt University School of Medicine, Nashville, TN 37232, USA; ^4^ Current address: Department of Cancer Biology, College of Medicine, University of Cincinnati, Cincinnati, OH 45221, USA

**Keywords:** bladder cancer, TKIs, RTKIs, COX-2, targeted therapies

## Abstract

Among challenges of targeted therapies is the activation of alternative pro-survival signaling pathways in cancer cells, resulting in an acquired drug resistance. Cyclooxygenase-2 (COX-2) is overexpressed in bladder cancer cells, making it an attractive molecular target for the detection and treatment of cancer. Fluorocoxib A is an optical imaging agent that selectively targets COX-2. In this study, we evaluated the ability of fluorocoxib A to monitor the responses of bladder cancer to targeted therapies *in vivo*. The effects of several tyrosine kinase inhibitors (TKIs: axitinib, AB1010, toceranib, imatinib, erlotinib, gefitinib, imatinib, sorafenib, vandetanib, SP600125, UO126, and AZD 5438) on COX-2 expression were validated in ten human and canine bladder cancer cell lines (J82, RT4, T24, UM-UC-3, 5637, SW780, TCCSUP, K9TCC#1Lillie, K9TCC#2Dakota, K9TCC#5Lilly) *in vitro*. The effects of TKIs on bladder cancer *in vivo* were evaluated using the COX-2-expressing K9TCC#5Lilly xenograft mouse model and detected by fluorocoxib A. The increased COX-2 expression was detected by all tested TKIs in at least one of the tested COX-2-expressing bladder cancer cell lines (5637, SW780, TCCSUP, K9TCC#1Lillie, K9TCC#2Dakota, and K9TCC#5Lilly) *in vitro*. In addition, fluorocoxib A uptake correlated with the AB1010- and imatinib-induced COX-2 expression in the K9TCC#5Lilly xenografts *in vivo*. In conclusion, these results indicate that fluorocoxib A could be used for the monitoring the early responses to targeted therapies in COX-2-expressing bladder cancer.

## INTRODUCTION

Bladder cancer is the 6th most common type of cancer in the United States and one of the most expensive malignancies to treat due to high recurrence rates and lack of improved treatment options over the past several decades [1–3]. In 90% of all cases, bladder cancer originates from the epithelial lining of the bladder known as the urothelium. This type of bladder cancer is known as the transitional cell carcinoma (TCC) or urothelial carcinoma [4]. Early detection of bladder cancer proves to provide better prognostic outcomes for patients diagnosed with bladder cancer [5]. However, despite demonstrating the need for improved diagnostic screening, prevention, and treatment options, bladder cancer still remains one of the most commonly diagnosed malignancies in the United States [6]. This drives the need for improved detection and novel therapeutic options for patients diagnosed with both non-muscle invasive bladder cancer and muscle invasive bladder cancer.

Receptor tyrosine kinases (RTKs) mediate key signaling pathways involved in cell proliferation, differentiation, survival, and cell migration [7, 8]. Mutations of RTKs can affect the expression of downstream signaling pathways such as the MAP kinase, PI3K/Akt, and cyclooxygenase (COX)-2 pathways, resulting in aberrant cell function, which plays an important role in a number of biological processes, including the progression of cancer [9]. RTKs, such as the c-Kit receptor, platelet derived growth factor receptor (PDGFR), and vascular endothelial growth factor receptor (VEGFR), are overexpressed in many types of cancer, including bladder cancer [10–12]. Inhibitors of RTKs (RTKIs) are therefore used as the targeted therapy options for patients diagnosed with cancer that overexpress RTKs to inhibit auto-phosphorylation and downstream signal transduction, halting tumor progression [13, 14]. For many decades, kinases have been extensively studied as the potential drug targets and to date, thirty eight RTKIs have been FDA-approved for the treatment of cancer, including imatinib (Gleevec) [15, 16]. There are even more RTKIs under development in pre-clinical research and clinical trial phases, including AB1010 (Masitinib^®^ or Masivet) [17].

Imatinib is a potent and selective inhibitor of the c-Kit (IC_50_ = 100 nM) and PDGFRα/β (IC_50_ = 100 nM) receptors [18, 19]. It is currently used as a first-line therapy option for a treatment of chronic myeloid leukemia (CML) and for advanced stage gastrointestinal stromal tumors (GIST) [20–22]. AB1010 is a novel RTKI that also selectively targets the c-Kit (IC_50_ = 200 nM) and PDGFRα/β (IC_50_ = 540 nM and 800 nM, respectively) receptors [17]. AB1010 is approved in the Europe to be used for treatment of canine mast cell tumors and has been investigated for the treatment of human patients diagnosed with GIST and pancreatic cancers alone or in combination with chemotherapy agents [23–28]. Both imatinib and AB1010 are well-tolerated with only a few side effects. However, a common challenge associated with targeted therapies is the activation of alternative pro-survival signaling pathways, resulting in acquired drug resistance. Previous studies indicate that treatment with chemotherapeutic agents and targeted therapies, such as RTKIs, increased COX-2 expression in bladder cancer cells, glioma cancer stem cells, non-small cell lung cancer, and oral squamous cell carcinoma cells *in vitro* [29–33]*.* This drives the need for monitoring the expression changes of key molecular targets in order to early detect responses to targeted therapies. This approach of precision-based cancer medicine may lead to improvement of prognostic outcomes in patients diagnosed with bladder cancer.

COX-2 is one of the key proteins responsible for promoting angiogenesis, cell proliferation, and inhibiting apoptosis [34–37]. COX-2 is overexpressed in many types of cancer, including bladder cancer, and is often an indicator of poor patient prognosis [38]. Overexpressed COX-2 in cancer can therefore be used as a target for the treatment and detection of bladder cancer [39–41]. To improve tumor detection during cystoscopy procedures, fluorescently-labeled contrast agents have been validated for the detection of bladder cancer. [42]. Currently, inhibitors of COX-2 (non-steroidal anti-inflammatory drugs, NSAIDs) are used both for the prevention and treatment of cancer, but recently have also been investigated as contrast imaging agents. Previously published studies indicate that fluorescently-labeled COX-2 inhibitors, which bind to the active site of COX-2, are suitable candidates for targeted optical imaging because of their stable properties, high specificity for the target protein (COX-2), and systemic route of administration [43–46]. Novel optical imaging agent, fluorocoxib A, is a rhodamine-conjugated analog of indomethacin that selectively targets COX-2-expressing cells [47]. This imaging agent has been extensively studied both *in vitro* and *in vivo* for the detection of cancer, demonstrating highly selective and specific uptake by COX-2-expressing cancer cells when compared to surrounding normal cells [40, 41, 48].

In this study, we evaluated the effects of several TKIs on the expression of COX-2 in human and canine bladder cancer cell lines *in vitro*. In addition, we have validated the ability of fluorocoxib A to detect and monitor the early responses to RTKIs treatment through induced COX-2 expression using the K9TCC#Lilly xenograft mouse model *in vivo*.

## RESULTS

### TKIs increased COX-2 expression in six out of ten tested bladder cancer cell lines

**Figure 1:**
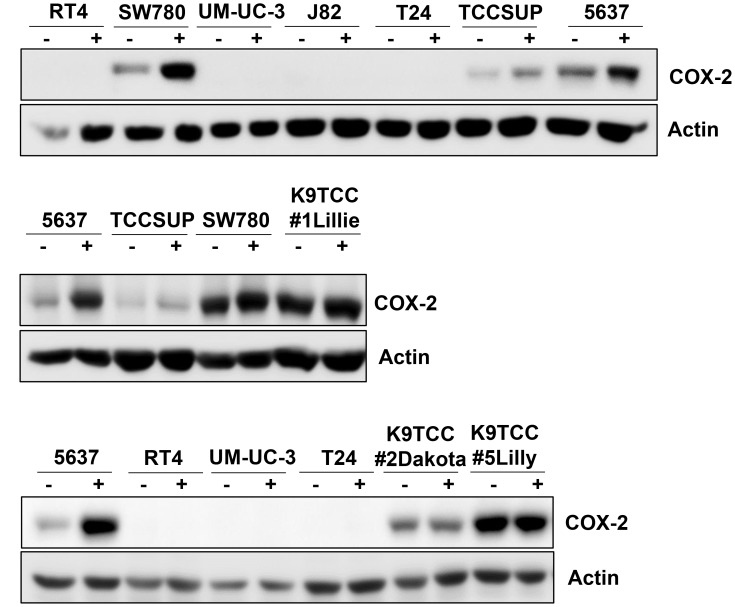
COX-2 expression in ten tested bladder cancer cell lines. Human bladder cancer J82, RT4, T24, UM-UC-3, 5637, SW780, and TCCSUP cells and canine bladder cancer K9TCC#1Lillie, K9TCC#5Lilly, and K9TCC#2Dakota cells were cultured in media with or without serum for 24 h. COX-2 expression was detected by WB analysis (*n* = 2). Actin was used as a loading control.

The basal levels of COX-2 expression in tested bladder cancer cells were confirmed by WB analysis as shown in Figure 1. Bladder Cancer cells were grown in media with or without serum for 24 hours. Six out of ten tested bladder cancer cell lines (5637, SW780, TCCSUP, K9TCC#1Lillie, K9TCC#2Dakota, and K9TCC#5Lilly) were positive for COX-2 expression. The highest COX-2 expression levels were detected in SW780, K9TCC#2Dakota, K9TCC#5Lilly, K9TCC#1Lillie cells, with the lowest expression level in TCCSUP cells. No COX-2 expression was detected in four out of ten bladder cancer cell lines (J82, RT4, T24, and UM-UC-3).

**Figure 2:**
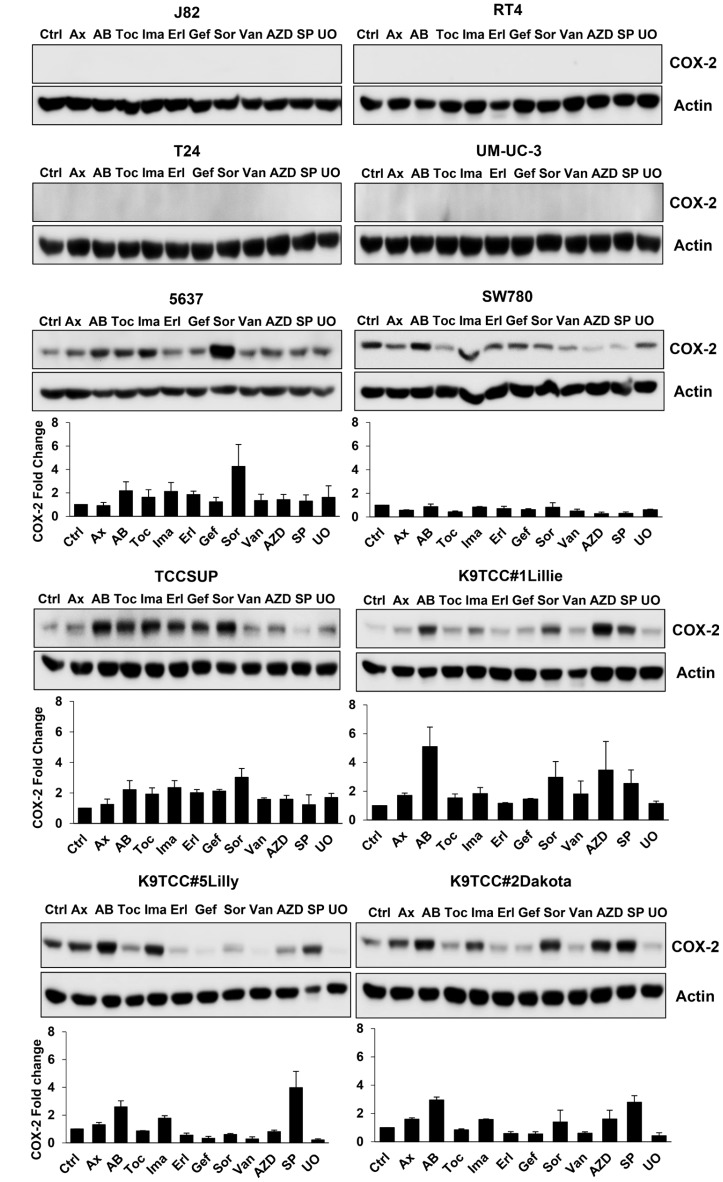
RTKIs and TKIs increased COX-2 expression in six out of ten tested bladder cancer cells. Human bladder cancer J82, RT4, T24, UM-UC-3, 5637, SW780, and TCCSUP cells and canine bladder cancer K9TCC#1Lillie, K9TCC#5Lilly, and K9TCC#2Dakota cells were treated with 5 μM dose of tested RTKIs and TKIs for 24 h. The expression of COX-2 was determined by WB analysis and actin was used as a loading control. Densitometry evaluation of COX-2/actin protein bands from WB analysis (*n* = 2) was performed using VisionWorks acquisition and analysis software (Analytik Jena). Densitometry analysis values represent mean ± standard error of fold change of COX-2 expression of each treatment to control from two independent experiments.

Our previously published studies demonstrated that treatments with RTKIs increase COX-2 expression in oral squamous cell carcinoma [32] and bladder cancer [29] cells *in vitro*. All tested RTKIs and TKIs at dose of 5 μM for 24 h induced COX-2 expression in at least one of the tested COX-2-expressing bladder cancer cell lines (5637, SW780, TCCSUP, K9TCC#1Lillie, K9TCC#2Dakota, and K9TCC#5Lilly) *in vitro* as shown in Figure 2. The highest 2–5-fold increase of COX-2 expression was detected by AB1010 in five out of six tested COX-2-expressing bladder cancer cell lines. Sorafenib increased COX-2 expression by 1.6–4-fold in four out of six and SP600125 increased COX-2 expression by 2.5–4-fold in three out of six tested COX-2-expressing bladder cancer cell lines. AZD5438, a cyclin-dependent kinase-1/2/9 inhibitor [49], increased COX-2 expression by 1.6–3.5-fold in two out of six tested COX-2-expressing bladder cancer cell lines. COX-2 expression levels were increased by 1.6–2-fold in two out of six tested cell lines by toceranib, 1.6–2-fold in five out of six tested cell lines by imatinib, 2-fold in two out of six tested cell lines by erlotinib, 2-fold in one out of six tested cell lines by gefitinib and vandetanib, 1.6–1.7-fold in two out of six tested cell lines by UO126, and 1.5–1.7-fold in all six tested cell lines by axitinib.

### AB1010 and imatinib increased COX-2 expression in tested COX-2-expressing bladder cancer cells in a dose-dependent manner

**Figure 3:**
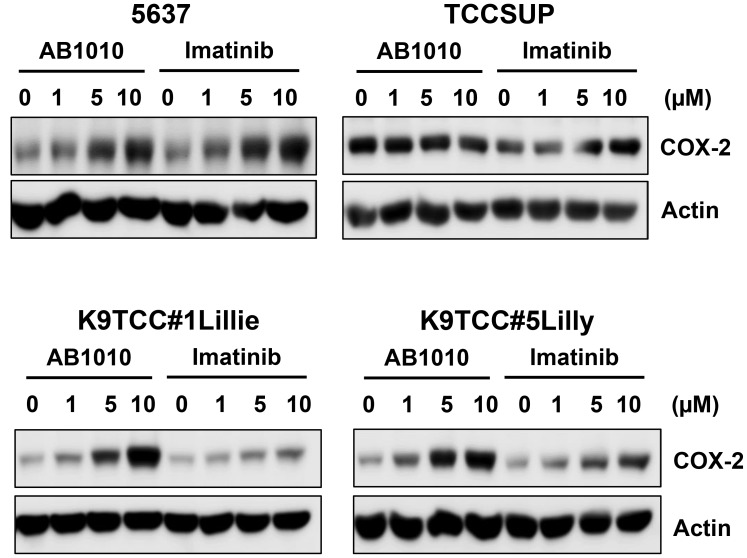
AB1010 and imatinib increased COX-2 expression in tested COX-2-expressing bladder cancer cells in a dose-dependent manner. Human bladder cancer 5637 and TCCSUP cells and canine bladder cancer K9TCC#1Lillie and K9TCC#5Lilly cells were treated with 1, 5, and 10 μM dose of AB1010 or imatinib for 24 h. COX-2 expression was determined by WB analysis (*n* = 2). Actin was used as a loading control.

We further investigated the effect of two RTKIs, AB1010 and imatinib, in four COX-2-expressing bladder cancer cell lines (5637, TCCSUP, K9TCC#1Lillie, and K9TCC#5Lilly). RTKIs, AB1010 and imatinib, selectively target the c-Kit and PDFGRα/β receptors [17–19]. Both RTKIs, AB1010 and imatinib, increased COX-2 expression in a dose-dependent manner in 5637, K9TCC#1Lillie, and K9TCC#5Lilly cells *in vitro* (Figure 3). Only imatinib, but not AB1010, increased COX-2 expression in a dose-dependent manner in TCCSUP cells *in vitro* (Figure 3).

The inhibition of the c-Kit and PDFGRβ receptors was confirmed by WB analysis as shown in Supplementary Figure 1.

### RTKIs-induced COX-2 expression in K9TCC#5Lilly xenograft tumors was detected by fluorocoxib A uptake

**Figure 4:**
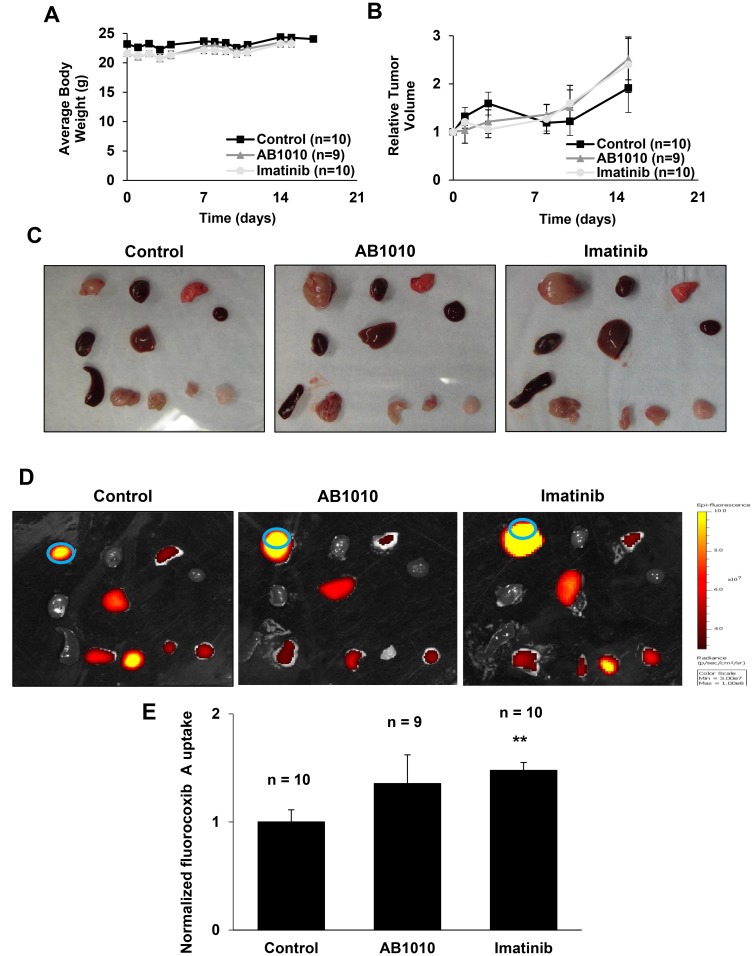
RTKIs-induced COX-2 expression in K9TCC#5Lilly xenograft tumors detected by fluorocoxib A uptake. (**A**) Averaged body weight and (**B**) relative tumor volume of mice over time from control, AB1010-, and imatinib-treated groups were plotted as shown. After a treatment, mice were injected with fluorocoxib A (1 mg/kg, s.c.) and imaged using the IVIS Lumina system. After sacrificing the mice, the dissected organs/tissues were (**C**) photographed and (**D**) imaged *ex vivo.* Organs/tissues from left to right: Row 1 - tumor, heart, lung; Row 2 - kidney, liver, blood; Row 3 - pancreas & spleen, small intestine, muscle, and fat. Blue circle depicts the region of interest (ROI) area used for IVIS image data analysis. (**E**) Relative average radiant efficiency values of fluorocoxib A uptake by the K9TCC#5Lilly xenograft tumors were normalized to blood. The values of tumor-to-noise ratio (TNR) from mice treated with AB1010 (*n* = 9) and imatinib (*n* = 10) were normalized to control group (*n*=10) and plotted as mean ± standard error. Student’s *t*-test was used for a statistical analysis, ^**^ *p* < 0.01.

Treatment with either RTKIs, AB1010 and imatinib, had no adverse events on the growth of the mice over time *in vivo* as no significant differences in body weights were observed between RTKIs-treated and control groups as shown in Figure 4A. After the first week of RTKIs treatment, a slight decrease in relative tumor volumes in AB1010- and imatinib-treated mice were detected when compared to relative tumor volumes of mice from the control group as shown in Figure 4B. However, after two weeks of RTKIs treatments, relative tumor volumes in mice from both treated groups increased when compared to volume of tumors in mice from control group.

Following RTKIs treatment for two weeks, fluorocoxib A was administered (1 mg/kg) to all mice. After 4 h of fluorocoxib A uptake, the mice were sacrificed, and the dissected tissues were photographed (Figure 4C) and imaged *ex vivo* using the Xenogen IVIS Lumina optical imaging system (Figure 4D). Specific uptake of fluorocoxib A was detected in the dissected COX-2-expressing K9TCC#5Lilly xenograft tumors. The increased fluorocoxib A uptake by a 1.4- and 1.5-fold was detected in xenograft tumors isolated from mice treated by AB1010 and Imatinib when compared to xenograft tumors isolated from control mice as shown in Figure 4E.

### RTKI-induced COX-2 expression in K9TCC#5Lilly xenograft tumors *in vivo*

**Figure 5:**
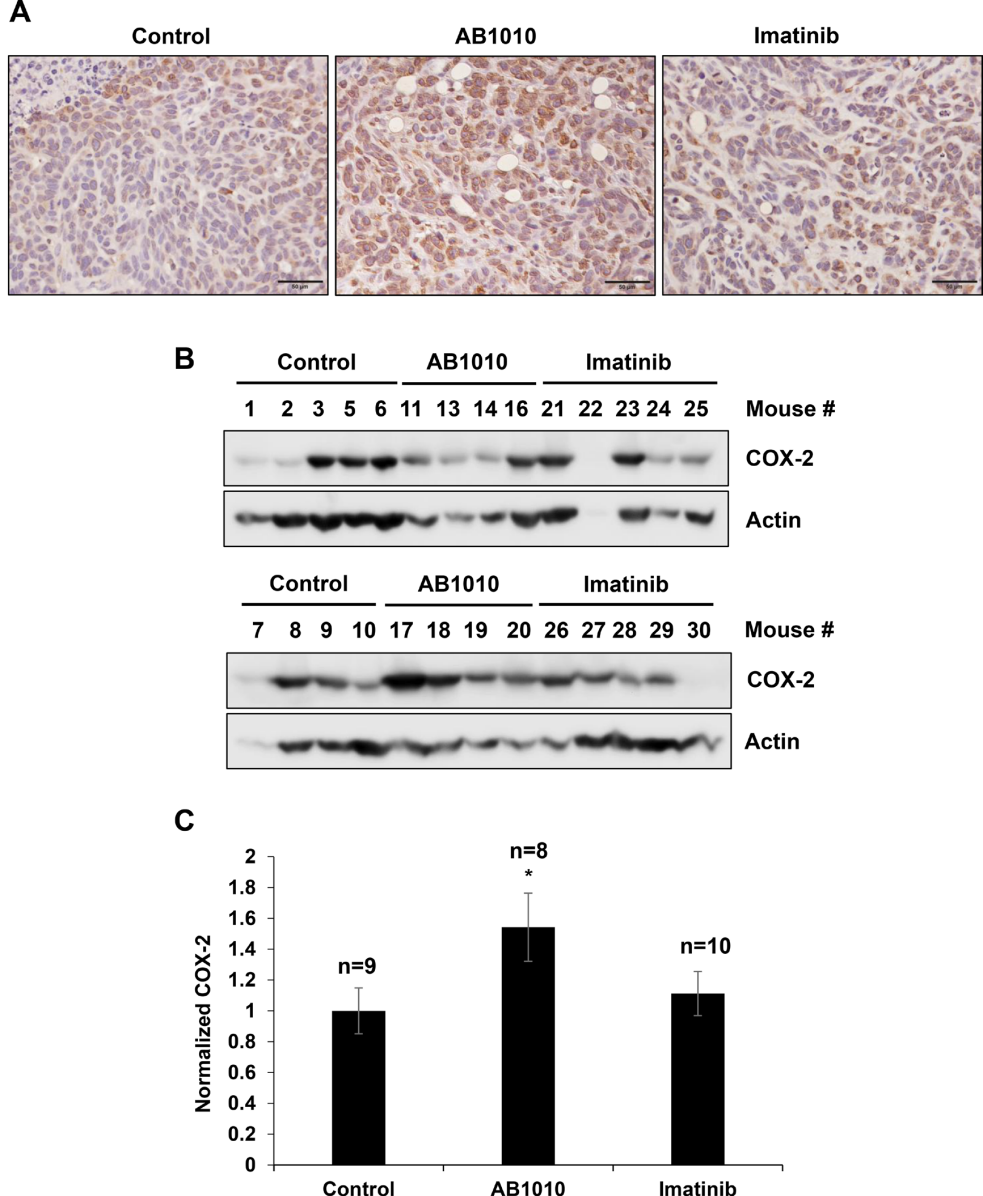
RTKIs-induced COX-2 expression in K9TCC#5Lilly xenograft tumors *in vivo*. The upregulation of COX-2 by AB1010 and imatinib in the K9TCC#5Lilly xenograft tumors was detected by (**A**) IHC and (**B**) WB analyses. Brown color indicates a positive staining for COX-2 protein expression in cells. Objective 20x, scale bar 50μm. Actin was used as a loading control for WB. (**C**) Densitometry evaluation of normalized COX-2 to actin expressions were performed using the VisionWorks acquisition (Analytik Jena) and analyzed by Image J software (NIH). Obtained values from AB1010 (*n* = 8) and imatinib (*n* = 10) treated groups were normalized to a control group (*n* = 9) and plotted as mean ± standard error from two independent WB gels that were run in two independent times (*n* = 4). Student’s *t*-test was used for a statistical analysis, ^*^ *p* < 0.05.

Tested RTKIs, AB1010 and imatinib, induced COX-2 expression in K9TCC#5Lilly cells not only *in vitro* (Figure 2 and Figure 3), but also in xenograft tumors detected in mice *in vivo* by IHC and WB analysis as shown in Figure 5A–5C, respectively. AB1010 and imatinib increased the COX-2 expression by 1.5-fold (*n* = 8 mice) and 1.1-fold (*n* = 10 mice), respectively, in K9TCC#5Lilly xenograft tumors as compared to tumors isolated from untreated mice (*n* = 9).

## DISCUSSION

Bladder cancer remains one of the most expensive malignancies to treat primarily due to high rates of recurrence [1]. In recent years, targeted therapies are commonly used as a treatment option for patients diagnosed with bladder cancer. However, a common challenge associated with targeted therapies is the activation of alternative pro-survival signaling pathways and induced receptor mutations, resulting in an acquired drug resistance [50–52]. Another challenge for the detection of bladder cancer, is that conventional optical imaging modalities have several limitations, including poor ability to detect bladder cancer at the early stages and poor differentiation of tumor margins during resection procedures [42]. These challenges drive the need for a better detection of bladder cancer and to improve the early detection of the tumor’s responses to treatment.

For decades, research has relied on numerous pre-clinical mostly rodent animal models to study the development, progression and treatment of cancer. More recently, companion animals (dogs and cats) with naturally occurring cancers have been utilized as valuable and still underutilized pre-clinical animal cancer models for the evaluation of novel therapeutic and diagnostic tools that have the potential to benefit human, in addition to the animal health. It has been determined that naturally occurring tumors of companion animals have many characteristics similar to human cancers including, genetic components (i.e., gene expression profile and intracellular signaling events) and common environmental factors responsible for the development/progression of the cancer [53]. Those common characteristics indicate that results from studies utilizing companion animals with spontaneously occurring tumors can be translated more effectively into human clinic to improve diagnostic and treatment options for human patients diagnosed with cancer [54–56]. The established and characterized canine and feline cancer cell lines serve as a valuable model to advance the validation of novel imaging and therapeutic agents for cancer [29, 32, 40, 57–60].

COX-2 plays a key role in promoting angiogenesis, cell proliferation, and inhibiting apoptosis [34–37, 61]. COX-2 is overexpressed in many types of cancer, including bladder cancer, and is often an indicator of poor prognosis in human [38, 62] as well in canine patients [63, 64]. In this study, we evaluated the effects of several RTKIs and TKIs on COX-2 expression in ten human and canine bladder cancer cell lines *in vitro*. Furthermore, the effects of the specific RTKIs, AB1010 and imatinib, on COX-2 expression in K9TCC#5Lilly xenograft tumors *in vivo* was also evaluated. COX-2 expression was detected in six out of ten tested bladder cancer cell lines (Figure 1). As shown in Figure 2, in the COX-2 positive bladder cancer cell lines, all tested RTKIs and TKIs increased COX-2 expression. AB1010 and imatinib selectively target the c-Kit and PDFGRα/β receptors, which are overexpressed in bladder cancer cells [17–19]. Despite inhibiting the phosphorylation of the PDGFRβ receptor (Supplementary Figure 1), AB1010 and imatinib increased COX-2 expression in a dose-dependent manner in the tested COX-2 positive bladder TCC cell lines (Figure 3). The results from our study are consistent with previously published studies where chemotherapeutic agents and targeted therapies increased COX-2 expression in bladder cancer cells, glioma cancer stem cells (CSCs), non-small cell lung cancer cells, and oral squamous cell carcinoma cells *in vitro* [29–33]*.* We have previously published that co-treatment of AB1010 with indomethacin abrogates the AB1010-induced COX-2 expression and inhibit cell growth of tested TCC cells *in vitro* [29]. This is also in agreement with a previously published study which determined that co-treatment of imatinib and celecoxib, was more effective than imatinib alone and sensitized imatinib-resistant K562 cells *in vitro* [65]*.*

The challenges associated with the detection of bladder cancer also contribute to the high bladder cancer recurrence rates, driving the need for improved diagnostic imaging techniques. To improve diagnostic imaging tools during cystoscopy procedures, a key focus in pre-clinical research is to evaluate the ability of fluorescently-labeled contrast agents for the detection and monitoring of bladder cancer [42, 66–68]. Previously published studies indicate that fluorescently-labeled COX-2 inhibitors, such as fluorocoxib A, are suitable candidates for targeted optical imaging because of their high specificity for the target protein, COX-2 [43–46]. Fluorocoxib A has been extensively studied both *in vitro* and *in vivo* for the detection of inflammation and cancer, demonstrating highly selective and specific uptake by COX-2-expressing tissues when compared to surrounding normal tissues [40, 41, 48, 69]. In this study, we validated the ability of fluorocoxib A to detect changes in COX-2 expression after treatment with the targeted TKIs therapies, AB1010 and imatinib *in vivo* using the COX-2-expressing K9TCC#5Lilly xenograft mouse model. The initial decrease in relative tumor volumes in mice treated with AB1010 and imatinib after first week of therapy, followed by the increase in relative tumor volume after two weeks of therapy, suggests possible acquired drug resistance to RTKIs. Higher uptake of fluorocoxib A was observed in the K9TCC#5Lilly xenograft tumors from mice treated with AB1010 and imatinib when compared to volume of tumors from untreated mice as shown in Figure 4. The RTKI-induced COX-2 expression in K9TCC#5Lilly xenograft tumors was confirmed by IHC (Figure 5A) and WB analysis (Figure 5B and 5C). The specific uptake and increased intensity of fluorocoxib A (Figure 4) by the K9TCC#5Lilly xenograft tumors correlated with the RTKI-induced COX-2 expression both *in vitro* (Figure 2 and 3) and *in vivo* (Figure 5). These results indicate that fluorocoxib A is highly sensitive and specific to detect and monitor the changes of COX-2 expression in bladder cancer. In an era of precision medicine, there is a rising importance to detect not only the basal levels but also monitor the changes in the expression of key molecular drivers of the tumor through the course of treatment to predict early responders from non-responders and to improve the outcomes of patients diagnosed with cancer.

In conclusion, the increased COX-2 expression was detected by all tested RTKIs and TKIs in at least one of the tested COX-2-expressing bladder TCC cell lines (5637, SW780, TCCSUP, K9TCC#1Lillie, K9TCC#2Dakota, and K9TCC#5Lilly) *in vitro*. In addition, fluorocoxib A uptake correlated with the AB1010- and imatinib-induced COX-2 expression detected by IHC and WB analysis in the K9TCC#5Lilly xenografts *in vivo*. In conclusion, these results indicate that fluorocoxib A could be used for the monitoring the tumor’s responses to targeted therapies in COX-2-expressing bladder cancers.

## MATERIALS AND METHODS

### Antibodies and reagents

The antibodies for p-c-Kit (Tyr721, sc-18077), p-PDGFRβ (F-10, sc-365464), PDGFRβ (11H4, sc-80991), COX-2 (C-20, sc-1745), actin (C-4, sc-47778), and secondary donkey anti-goat (sc-2020) were purchased from Santa Cruz Biotechnology (Santa Cruz, CA, USA); antibody for COX-2 (aa 570-598, 160106) was purchased from Cayman Chemical (Ann Arbor, MI, USA); antibody for c-Kit (961-976, PC34) was purchased from Millipore Sigma (Burlington, MA, USA); and secondary anti-rabbit (cs-7074) antibody were purchased from Cell Signaling Technology (Danvers, MA, USA). AZD 5438 (AZD) and toceranib (Toc) were purchased from Tocris Bioscience (Minneapolis, MN, USA). Erlotinib (Erl), gefitinib (Gef), imatinib (Ima), sorafenib (Sor), SP600125 (SP), vandetanib (Van), and UO126 (UO) were purchased from Cell Signaling Technology. Axitinib (Ax) was purchased from Millipore Sigma (Burlington, MA, USA); and AB1010 (AB, Masitinib^®^ or Masivet) was purchased from ApexBio (Boston, MA, USA). All other chemicals and reagents were purchased from Thermo Fisher Scientific (Pittsburgh, PA, USA), unless otherwise specified.

### Cell lines

Human bladder cancer cell lines J82, RT4, T24, UM-UC-3, 5637, SW780, and TCCSUP were purchased from American Type Culture Collection (ATCC, Manassas, VA, USA). Cell lines were authenticated via short tandem repeat (STR) DNA profiling by Genetica DNA laboratories (Burlington, NC, USA). J82 and TCCSUP cells were grown in MEM media supplemented with MEM non-essential amino acids and sodium pyruvate; RT4 and T24 cells were grown in McCoy’s media; UM-UC-3 cells were grown in MEM media; and 5637 and SW780 cells were grown in RPMI-1640 media. All culture media were supplemented with 10% fetal bovine serum, 100 I.U. penicillin, and 100 μg/mL streptomycin. Canine bladder transitional cell carcinoma (K9TCC) cell lines K9TCC#1Lillie, K9TCC#2Dakota, and K9TCC#5Lilly were established and characterized in the laboratory of Dr. Cekanova as described previously [58]. The K9TCC cell lines were grown in a complete RPMI-1640 medium supplemented with 10% fetal bovine serum, 100 I.U. penicillin, and 100 μg/mL streptomycin.

### Animals

All animal experiments were performed in accordance with approved UTK IACUC protocols. Thirty 5-week-old athymic female nude mice (Charles River, Boston, MA, USA) were randomly divided into three groups (*n* = 10/group). The COX-2 positive bladder cancer K9TCC#5Lilly cells were injected s ubcutaneously (s.c.) at a density of 1.7–2.0 × 10^6^ cells mixed with a 1:1 ratio of PBS and Matrigel. After 2 weeks of tumor development, treatment with the RTKIs, AB1010 and imatinib, was initiated. Group 1 served as the control group of mice that received the vehicle (DMSO+0.9% NaCl). The other two groups of mice received the RTKI treatments, where Group 2 received AB1010 (3.75 mg/kg) and Group 3 received imatinib (7.5 mg/kg), three times per week for two weeks intraperitoneal (i.p.). Tumor volume was calculated as: V(mm^3^) = length(mm) × width(mm)^2^ × 0.52.

### Optical imaging *in vivo*

After the final RTKI treatments, fluorocoxib A was administered (1 mg/kg, s.c.) to all mice. Four hours after fluorocoxib A administration, the mice were sacrificed, tumor and other organs were dissected, and imaged *ex vivo* using the Xenogen IVIS Lumina optical imaging system with DsRed filters with excitation 500 to 550 nm, emission 575 to 650 nm, and bac kground 460 to 490 nm (Perkin Elmer, Waltham, MA, USA). The obtained total flux (p/s) and average radiant efficiency ([p/s/cm^²^/sr] / [μW/cm^²^]) of labeled regions of interest (ROI) of dissected tumor and other organs/tissues (heart, lung, kidney, liver, blood, spleen, pancreas, small intestine, muscle, and fat) were evaluated. Tumor-to-noise ratio (TNR) was calculated using the following equation: TNR = (tumor radiant efficiency values)/(blood radiant efficiency values). The TNR for each treatment groups (AB1010 and imatinib) were normalized to Group 1 (control). Each dissected tumor was divided into pieces; one piece of tumor was fixed in a 10% neutral buffered-formalin for histology and immunohistochemistry (IHC) analysis. Another piece of tumor was kept in RNAlater solution and stored at −80° C freezer until western blotting (WB) analyses were performed.

### Immunohistochemistry (IHC)

Dissected tissues from mice were formalin-fixed, paraffin-embedded, and sectioned at 7 μm. Hematoxylin and eosin (H&E) staining was performed following a standard histological staining protocol. The IHC staining was performed as described previously [41]. After de-paraffinization, the antigen retrieval using sodium citrate pH 6.0 was performed for 20 min in the antigen retriever (Electron Microscopy Sciences, Hatfield, PA, USA). The blocking of endogenous peroxidase activity was performed by 3% H^2^O^2^ in 50% methanol for 5 min, followed by the blocking of non-specific signal using protein block solution (BioGenex, Fremont, CA, USA). Tissues were incubated with the COX-2 primary antibody (Cayman), followed by the incubation with the specific biotinylated secondary antibodies, streptavidin/HRP detection system, and visualized by 3,3'-diaminobenzidine (DAB) staining. Nuclei were counter-stained with a hematoxylin and slides were evaluated using a Leitz DMRB microscope (Leica). The images were captured by a DP73 camera (Hunt Optics and Imaging, Pittsburgh, PA, USA) using CellSens Standard software (Olympus, Center Valley, PA, USA).

### Western blotting analysis

The tested bladder cancer cells were seeded in 10 cm tissue culture dishes at a concentration of 2 × 10^6^ cells/dish in complete media. After 24 h incubation, cells were treated with 5 μM concentration of the tested RTKIs (as shown in Figure 2) and with 0, 1, 5, and 10 μM of AB1010 and imatinib (as shown in Figure 3) in complete media for an additional 24 h. DMSO was used as the control. The cell lysates and tissue samples were lysed in ice-cold RIPA buffer supplemented with protease and phosphatase inhibitors cocktail (1 mM PMSF; 1 μg/ml aprotinin; 1 μg/ml leupeptin; 5 mM Na_3_VO_4_; 5 mM NaF) and briefly sonicated on ice. Protein concentrations were measured using Pierce^®^ BCA protein assay (Thermo Scientific, Rockford, IL, USA). Equal amounts of proteins were loaded onto SDS-PAGE gels and transferred to nitrocellulose membranes. After blocking, the membranes were incubated with primary antibodies overnight at 4° C, followed by incubation with horseradish peroxidase-conjugated secondary antibodies for 1 h at room temperature. The immuno-reactive bands were visualized using the ECL prime chemiluminescence system (GE Healthcare Life Sciences, Marlborough, MA, USA) and the images were captured using the BioSpectrum^®^ 815 imaging system (Analytik Jena, Upland, CA, USA). Densitometry analysis was performed using the VisionWorks acquisition (Analytik Jena) and analysis software (Analytik Jena and Image J, NIH).

### Statistical analysis

Statistical analysis was conducted using the Student’s *t-*test to establish significant differences among treatment groups. Results were considered statistically significant at ^*^ *p* < 0.05 and ^**^ *p* < 0.01.

## SUPPLEMENTARY MATERIALS



## Abbreviations

COX-2: cyclooxygenase-2
IHC: immunohistochemistry
Fluorocoxib A: a N-[(5-carboxy-X-rhodaminyl)but-4-yl]-2-[1-(4-chlorobenzoyl)-5-methoxy-2-methyl-1H-indol-3-yl]acetamide
TCC: transitional cell carcinoma
RTKIs: receptor tyrosine kinase inhibitors
TKI: tyrosine kinase inhibitors
TNR: Tumor-to-noise ratio
WB: western blotting
WLC: white light cystoscopy
PDGFR: platelet derived growth factor receptor
VEGFR: vascular endothelial growth factor receptor

## References

[1] Institute NC SEER Cancer Stat Facts: Bladder Cancer Bethesda MD (ed.), 2018.

[2] Svatek RS, Hollenbeck BK, Holmäng S, Lee R, Kim SP, Stenzl A, Lotan Y The economics of bladder cancer: costs and considerations of caring for this disease. Eur Urol. 2014; 66:253–62. 10.1016/j.eururo.2014.01.006. 24472711

[3] Botteman MF, Pashos CL, Redaelli A, Laskin B, Hauser R The health economics of bladder cancer: a comprehensive review of the published literature. Pharmacoeconomics. 2003; 21:1315–30. 10.1007/BF03262330. 14750899

[4] Society AC Bladder Cancer Stages In: Eidsmoe K (ed.), 2019.

[5] Jacobs BL, Lee CT, Montie JE Bladder cancer in 2010: how far have we come? CA Cancer J Clin. 2010; 60:244–72. 10.3322/caac.20077. 20566675

[6] Morris DS, Weizer AZ, Ye Z, Dunn RL, Montie JE, Hollenbeck BK Understanding bladder cancer death: tumor biology versus physician practice. Cancer. 2009; 115:1011–20. 10.1002/cncr.24136. 19152434

[7] Ullrich A, Schlessinger J Signal transduction by receptors with tyrosine kinase activity. Cell. 1990; 61:203–12. 10.1016/0092-8674(90)90801-K. 2158859

[8] Lemmon MA, Schlessinger J Cell signaling by receptor tyrosine kinases. Cell. 2010; 141:1117–34. 10.1016/j.cell.2010.06.011. 20602996 PMC2914105

[9] Blume-Jensen P, Hunter T Oncogenic kinase signalling. Nature. 2001; 411:355–65. 10.1038/35077225. 11357143

[10] Cancer Genome Atlas Research Network Comprehensive molecular characterization of urothelial bladder carcinoma. Nature. 2014; 507:315–22. 10.1038/nature12965. 24476821 PMC3962515

[11] Crew JP Vascular endothelial growth factor: an important angiogenic mediator in bladder cancer. Eur Urol. 1999; 35:2–8. 10.1159/000019811. 9933788

[12] Pan CX, Yang XJ, Lopez-Beltran A, MacLennan GT, Eble JN, Koch MO, Jones TD, Lin H, Nigro K, Papavero V, Tretiakova M, Cheng L c-kit Expression in small cell carcinoma of the urinary bladder: prognostic and therapeutic implications. Mod Pathol. 2005; 18:320–23. 10.1038/modpathol.3800318. 15502806

[13] Arora A, Scholar EM Role of tyrosine kinase inhibitors in cancer therapy. J Pharmacol Exp Ther. 2005; 315:971–79. 10.1124/jpet.105.084145. 16002463

[14] Hartmann JT, Haap M, Kopp HG, Lipp HP Tyrosine kinase inhibitors - a review on pharmacology, metabolism and side effects. Curr Drug Metab. 2009; 10:470–81. 10.2174/138920009788897975. 19689244

[15] Nagar B, Bornmann WG, Pellicena P, Schindler T, Veach DR, Miller WT, Clarkson B, Kuriyan J Crystal structures of the kinase domain of c-Abl in complex with the small molecule inhibitors PD173955 and imatinib (STI-571). Cancer Res. 2002; 62:4236–43. 12154025

[16] Bhullar KS, Lagarón NO, McGowan EM, Parmar I, Jha A, Hubbard BP, Rupasinghe HP Kinase-targeted cancer therapies: progress, challenges and future directions. Mol Cancer. 2018; 17:48. 10.1186/s12943-018-0804-2. 29455673 PMC5817855

[17] Dubreuil P, Letard S, Ciufolini M, Gros L, Humbert M, Castéran N, Borge L, Hajem B, Lermet A, Sippl W, Voisset E, Arock M, Auclair C, et al Masitinib (AB1010), a potent and selective tyrosine kinase inhibitor targeting KIT. PLoS One. 2009; 4:e7258. 10.1371/journal.pone.0007258. 19789626 PMC2746281

[18] Heinrich MC, Griffith DJ, Druker BJ, Wait CL, Ott KA, Zigler AJ Inhibition of c-kit receptor tyrosine kinase activity by STI 571, a selective tyrosine kinase inhibitor. Blood. 2000; 96:925–32. 10910906

[19] Buchdunger E, Cioffi CL, Law N, Stover D, Ohno-Jones S, Druker BJ, Lydon NB Abl protein-tyrosine kinase inhibitor STI571 inhibits *in vitro* signal transduction mediated by c-kit and platelet-derived growth factor receptors. J Pharmacol Exp Ther. 2000; 295:139–45. 10991971

[20] Bauer S, Hagen V, Pielken HJ, Bojko P, Seeber S, Schütte J Imatinib mesylate therapy in patients with gastrointestinal stromal tumors and impaired liver function. Anticancer Drugs. 2002; 13:847–49. 10.1097/00001813-200209000-00010. 12394270

[21] Deininger MW, Druker BJ Specific targeted therapy of chronic myelogenous leukemia with imatinib. Pharmacol Rev. 2003; 55:401–23. 10.1124/pr.55.3.4. 12869662

[22] Vigneri P, Wang JY Induction of apoptosis in chronic myelogenous leukemia cells through nuclear entrapment of BCR-ABL tyrosine kinase. Nat Med. 2001; 7:228–34. 10.1038/84683. 11175855

[23] Hahn KA, Ogilvie G, Rusk T, Devauchelle P, Leblanc A, Legendre A, Powers B, Leventhal PS, Kinet JP, Palmerini F, Dubreuil P, Moussy A, Hermine O Masitinib is safe and effective for the treatment of canine mast cell tumors. J Vet Intern Med. 2008; 22:1301–09. 10.1111/j.1939-1676.2008.0190.x. 18823406

[24] Hahn KA, Legendre AM, Shaw NG, Phillips B, Ogilvie GK, Prescott DM, Atwater SW, Carreras JK, Lana SE, Ladue T, Rusk A, Kinet JP, Dubreuil P, et al Evaluation of 12- and 24-month survival rates after treatment with masitinib in dogs with nonresectable mast cell tumors. Am J Vet Res. 2010; 71:1354–61. 10.2460/ajvr.71.11.1354. 21034327

[25] Humbert M, Castéran N, Letard S, Hanssens K, Iovanna J, Finetti P, Bertucci F, Bader T, Mansfield CD, Moussy A, Hermine O, Dubreuil P Masitinib combined with standard gemcitabine chemotherapy: *in vitro* and *in vivo* studies in human pancreatic tumour cell lines and ectopic mouse model. PLoS One. 2010; 5:e9430. 10.1371/journal.pone.0009430. 20209107 PMC2832006

[26] Mitry E, Hammel P, Deplanque G, Mornex F, Levy P, Seitz JF, Moussy A, Kinet JP, Hermine O, Rougier P, Raymond E Safety and activity of masitinib in combination with gemcitabine in patients with advanced pancreatic cancer. Cancer Chemother Pharmacol. 2010; 66:395–403. 10.1007/s00280-010-1299-8. 20364428

[27] Deplanque G, Demarchi M, Hebbar M, Flynn P, Melichar B, Atkins J, Nowara E, Moyé L, Piquemal D, Ritter D, Dubreuil P, Mansfield CD, Acin Y, et al A randomized, placebo-controlled phase III trial of masitinib plus gemcitabine in the treatment of advanced pancreatic cancer. Ann Oncol. 2015; 26:1194–200. 10.1093/annonc/mdv133. 25858497 PMC4516046

[28] Adenis A, Blay JY, Bui-Nguyen B, Bouché O, Bertucci F, Isambert N, Bompas E, Chaigneau L, Domont J, Ray-Coquard I, Blésius A, Van Tine BA, Bulusu VR, et al Masitinib in advanced gastrointestinal stromal tumor (GIST) after failure of imatinib: a randomized controlled open-label trial. Ann Oncol. 2014; 25:1762–69. 10.1093/annonc/mdu237. 25122671 PMC4143095

[29] Bourn J, Cekanova M Cyclooxygenase inhibitors potentiate receptor tyrosine kinase therapies in bladder cancer cells *in vitro* . Drug Des Devel Ther. 2018; 12:1727–42. 10.2147/DDDT.S158518. 29942116 PMC6005335

[30] Kurtova AV, Xiao J, Mo Q, Pazhanisamy S, Krasnow R, Lerner SP, Chen F, Roh TT, Lay E, Ho PL, Chan KS Blocking PGE2-induced tumour repopulation abrogates bladder cancer chemoresistance. Nature. 2015; 517:209–13. 10.1038/nature14034. 25470039 PMC4465385

[31] Ma HI, Chiou SH, Hueng DY, Tai LK, Huang PI, Kao CL, Chen YW, Sytwu HK Celecoxib and radioresistant glioblastoma-derived CD133+ cells: improvement in radiotherapeutic effects. Laboratory investigation. J Neurosurg. 2011; 114:651–62. 10.3171/2009.11.JNS091396. 21054139

[32] Rathore K, Alexander M, Cekanova M Piroxicam inhibits Masitinib-induced cyclooxygenase 2 expression in oral squamous cell carcinoma cells *in vitro* . Transl Res. 2014; 164:158–68. 10.1016/j.trsl.2014.02.002. 24631063

[33] Altorki NK, Port JL, Zhang F, Golijanin D, Thaler HT, Duffield-Lillico AJ, Subbaramaiah K, Dannenberg AJ Chemotherapy induces the expression of cyclooxygenase-2 in non-small cell lung cancer. Clin Cancer Res. 2005; 11:4191–97. 10.1158/1078-0432.CCR-05-0108. 15930356

[34] Leahy KM, Ornberg RL, Wang Y, Zweifel BS, Koki AT, Masferrer JL Cyclooxygenase-2 inhibition by celecoxib reduces proliferation and induces apoptosis in angiogenic endothelial cells *in vivo* . Cancer Res. 2002; 62:625–31. 11830509

[35] Gately S, Li WW Multiple roles of COX-2 in tumor angiogenesis: a target for antiangiogenic therapy. Semin Oncol. 2004; 31:2–11. 10.1053/j.seminoncol.2004.03.040. 15179620

[36] Gakis G The role of inflammation in bladder cancer. Adv Exp Med Biol. 2014; 816:183–96. 10.1007/978-3-0348-0837-8_8. 24818724

[37] Qayyum T, McArdle P, Hilmy M, Going J, Orange C, Seywright M, Horgan P, Underwood M, Edwards J A prospective study of the role of inflammation in bladder cancer. Curr Urol. 2013; 6:189–93. 10.1159/000343537. 24917741 PMC3783284

[38] Kömhoff M, Guan Y, Shappell HW, Davis L, Jack G, Shyr Y, Koch MO, Shappell SB, Breyer MD Enhanced expression of cyclooxygenase-2 in high grade human transitional cell bladder carcinomas. Am J Pathol. 2000; 157:29–35. 10.1016/S0002-9440(10)64513-0. 10880372 PMC1850211

[39] Chen Z, Krishnamachary B, Penet MF, Bhujwalla ZM Acid-degradable Dextran as an Image Guided siRNA Carrier for COX-2 Downregulation. Theranostics. 2018; 8:1–12. 10.7150/thno.21052. 29290789 PMC5743456

[40] Cekanova M, Uddin MJ, Bartges JW, Callens A, Legendre AM, Rathore K, Wright L, Carter A, Marnett LJ Molecular imaging of cyclooxygenase-2 in canine transitional cell carcinomas *in vitro* and *in vivo* . Cancer Prev Res (Phila). 2013; 6:466–76. 10.1158/1940-6207.CAPR-12-0358. 23531445 PMC3671760

[41] Cekanova M, Uddin MJ, Legendre AM, Galyon G, Bartges JW, Callens A, Martin-Jimenez T, Marnett LJ Single-dose safety and pharmacokinetic evaluation of fluorocoxib A: pilot study of novel cyclooxygenase-2-targeted optical imaging agent in a canine model. J Biomed Opt. 2012; 17:116002. 10.1117/1.JBO.17.11.116002. 23117797 PMC3484194

[42] Jichlinski P, Leisinger HJ Fluorescence cystoscopy in the management of bladder cancer: a help for the urologist! Urol Int. 2005; 74:97–101. 10.1159/000083277. 15756058

[43] Schuller HM, Kabalka G, Smith G, Mereddy A, Akula M, Cekanova M Detection of overexpressed COX-2 in precancerous lesions of hamster pancreas and lungs by molecular imaging: implications for early diagnosis and prevention. ChemMedChem. 2006; 1:603–10. 10.1002/cmdc.200500032. 16892400

[44] Uddin MJ, Crews BC, Ghebreselasie K, Tantawy MN, Marnett LJ [I]-Celecoxib Analogues as SPECT Tracers of Cyclooxygenase-2 in Inflammation. ACS Med Chem Lett. 2011; 2:160–64. 10.1021/ml100232q. 21318094 PMC3037034

[45] Bhardwaj A, Kaur J, Sharma SK, Huang Z, Wuest F, Knaus EE Hybrid fluorescent conjugates of COX-2 inhibitors: search for a COX-2 isozyme imaging cancer biomarker. Bioorg Med Chem Lett. 2013; 23:163–68. 10.1016/j.bmcl.2012.10.131. 23200247

[46] Bhardwaj A, Kaur J, Wuest F, Knaus EE Fluorophore-labeled cyclooxygenase-2 inhibitors for the imaging of cyclooxygenase-2 overexpression in cancer: synthesis and biological studies. ChemMedChem. 2014; 9:109–16. 10.1002/cmdc.201300355. 24376205

[47] Uddin MJ, Crews BC, Blobaum AL, Kingsley PJ, Gorden DL, McIntyre JO, Matrisian LM, Subbaramaiah K, Dannenberg AJ, Piston DW, Marnett LJ Selective visualization of cyclooxygenase-2 in inflammation and cancer by targeted fluorescent imaging agents. Cancer Res. 2010; 70:3618–27. 10.1158/0008-5472.CAN-09-2664. 20430759 PMC2864539

[48] Ra H, González-González E, Uddin MJ, King BL, Lee A, Ali-Khan I, Marnett LJ, Tang JY, Contag CH Detection of non-melanoma skin cancer by *in vivo* fluorescence imaging with fluorocoxib A. Neoplasia. 2015; 17:201–07. 10.1016/j.neo.2014.12.009. 25748239 PMC4351298

[49] Byth KF, Thomas A, Hughes G, Forder C, McGregor A, Geh C, Oakes S, Green C, Walker M, Newcombe N, Green S, Growcott J, Barker A, Wilkinson RW AZD5438, a potent oral inhibitor of cyclin-dependent kinases 1, 2, and 9, leads to pharmacodynamic changes and potent antitumor effects in human tumor xenografts. Mol Cancer Ther. 2009; 8:1856–66. 10.1158/1535-7163.MCT-08-0836. 19509270

[50] Antonescu CR, Besmer P, Guo T, Arkun K, Hom G, Koryotowski B, Leversha MA, Jeffrey PD, Desantis D, Singer S, Brennan MF, Maki RG, DeMatteo RP Acquired resistance to imatinib in gastrointestinal stromal tumor occurs through secondary gene mutation. Clin Cancer Res. 2005; 11:4182–90. 10.1158/1078-0432.CCR-04-2245. 15930355

[51] Gajiwala KS, Wu JC, Christensen J, Deshmukh GD, Diehl W, DiNitto JP, English JM, Greig MJ, He YA, Jacques SL, Lunney EA, McTigue M, Molina D, et al KIT kinase mutants show unique mechanisms of drug resistance to imatinib and sunitinib in gastrointestinal stromal tumor patients. Proc Natl Acad Sci USA. 2009; 106:1542–47. 10.1073/pnas.0812413106. 19164557 PMC2635778

[52] Verweij J, Casali PG, Zalcberg J, LeCesne A, Reichardt P, Blay JY, Issels R, van Oosterom A, Hogendoorn PC, Van Glabbeke M, Bertulli R, Judson I Progression-free survival in gastrointestinal stromal tumours with high-dose imatinib: randomised trial. Lancet. 2004; 364:1127–34. 10.1016/S0140-6736(04)17098-0. 15451219

[53] MacEwen EG Spontaneous tumors in dogs and cats: models for the study of cancer biology and treatment. Cancer Metastasis Rev. 1990; 9:125–36. 10.1007/BF00046339. 2253312

[54] Rowell JL, McCarthy DO, Alvarez CE Dog models of naturally occurring cancer. Trends Mol Med. 2011; 17:380–88. 10.1016/j.molmed.2011.02.004. 21439907 PMC3130881

[55] Paoloni M, Khanna C Translation of new cancer treatments from pet dogs to humans. Nat Rev Cancer. 2008; 8:147–56. 10.1038/nrc2273. 18202698

[56] Cekanova M, Rathore K Animal models and therapeutic molecular targets of cancer: utility and limitations. Drug Des Devel Ther. 2014; 8:1911–21. 10.2147/DDDT.S49584. 25342884 PMC4206199

[57] Shapiro SG, Knapp DW, Breen M A cultured approach to canine urothelial carcinoma: molecular characterization of five cell lines. Canine Genet Epidemiol. 2015; 2:15. 10.1186/s40575-015-0028-3. 26401343 PMC4579363

[58] Rathore K, Cekanova M Animal model of naturally occurring bladder cancer: characterization of four new canine transitional cell carcinoma cell lines. BMC Cancer. 2014; 14:465. 10.1186/1471-2407-14-465. 24964787 PMC4082678

[59] Rathore K, Cekanova M A novel derivative of doxorubicin, AD198, inhibits canine transitional cell carcinoma and osteosarcoma cells *in vitro* . Drug Des Devel Ther. 2015; 9:5323–35. 10.2147/DDDT.S90859. 26451087 PMC4590339

[60] Smolensky D, Rathore K, Bourn J, Cekanova M Inhibition of the PI3K/AKT Pathway Sensitizes Oral Squamous Cell Carcinoma Cells to Anthracycline-Based Chemotherapy *In Vitro* . J Cell Biochem. 2017; 118:2615–24. 10.1002/jcb.25747. 27649518 PMC5572634

[61] Stasinopoulos I, Shah T, Penet MF, Krishnamachary B, Bhujwalla ZM COX-2 in cancer: gordian knot or Achilles heel? Front Pharmacol. 2013; 4:34. 10.3389/fphar.2013.00034. 23579438 PMC3619664

[62] Mohammed SI, Knapp DW, Bostwick DG, Foster RS, Khan KN, Masferrer JL, Woerner BM, Snyder PW, Koki AT Expression of cyclooxygenase-2 (COX-2) in human invasive transitional cell carcinoma (TCC) of the urinary bladder. Cancer Res. 1999; 59:5647–50. 10582676

[63] Mohammed SI, Khan KN, Sellers RS, Hayek MG, DeNicola DB, Wu L, Bonney PL, Knapp DW Expression of cyclooxygenase-1 and 2 in naturally-occurring canine cancer. Prostaglandins Leukot Essent Fatty Acids. 2004; 70:479–83. 10.1016/j.plefa.2003.10.002. 15062852

[64] Pestili de Almeida EM, Piché C, Sirois J, Doré M Expression of cyclo-oxygenase-2 in naturally occurring squamous cell carcinomas in dogs. J Histochem Cytochem. 2001; 49:867–75. 10.1177/002215540104900707. 11410611

[65] Arunasree KM, Roy KR, Anilkumar K, Aparna A, Reddy GV, Reddanna P Imatinib-resistant K562 cells are more sensitive to celecoxib, a selective COX-2 inhibitor: role of COX-2 and MDR-1. Leuk Res. 2008; 32:855–64. 10.1016/j.leukres.2007.11.007. 18083230

[66] Fradet Y, Grossman HB, Gomella L, Lerner S, Cookson M, Albala D, Droller MJ, Group PB, and PC B302/01 Study Group A comparison of hexaminolevulinate fluorescence cystoscopy and white light cystoscopy for the detection of carcinoma *in situ* in patients with bladder cancer: a phase III, multicenter study. J Urol. 2007; 178:68–73. 10.1016/j.juro.2007.03.028. 17499291

[67] Grossman HB, Gomella L, Fradet Y, Morales A, Presti J, Ritenour C, Nseyo U, Droller MJ, Group PB, and PC B302/01 Study Group A phase III, multicenter comparison of hexaminolevulinate fluorescence cystoscopy and white light cystoscopy for the detection of superficial papillary lesions in patients with bladder cancer. J Urol. 2007; 178:62–67. 10.1016/j.juro.2007.03.034. 17499283

[68] Kriegmair M, Zaak D, Rothenberger KH, Rassweiler J, Jocham D, Eisenberger F, Tauber R, Stenzl A, Hofstetter A Transurethral resection for bladder cancer using 5-aminolevulinic acid induced fluorescence endoscopy versus white light endoscopy. J Urol. 2002; 168:475–78. 10.1016/S0022-5347(05)64661-7. 12131291

[69] Uddin MJ, Werfel TA, Crews BC, Gupta MK, Kavanaugh TE, Kingsley PJ, Boyd K, Marnett LJ, Duvall CL Fluorocoxib A loaded nanoparticles enable targeted visualization of cyclooxygenase-2 in inflammation and cancer. Biomaterials. 2016; 92:71–80. 10.1016/j.biomaterials.2016.03.028. 27043768 PMC4833621

