# Supplementary material for: Detection of tyrosine kinase inhibitors-induced COX-2 expression in bladder cancer by fluorocoxib A
Source: Oncotarget. 2019 Aug 27;10(50):5168–80. doi: 10.18632/oncotarget.27125 (PMC6718263; doi:10.18632/oncotarget.27125)
Supplement: Supplementary file 1 [file oncotarget-10-5168-s001.pdf]

Detection of tyrosine kinase inhibitors-induced COX-2 expression in bladder cancer by fluorocoxib A

SUPPLEMENTARY MATERIALS

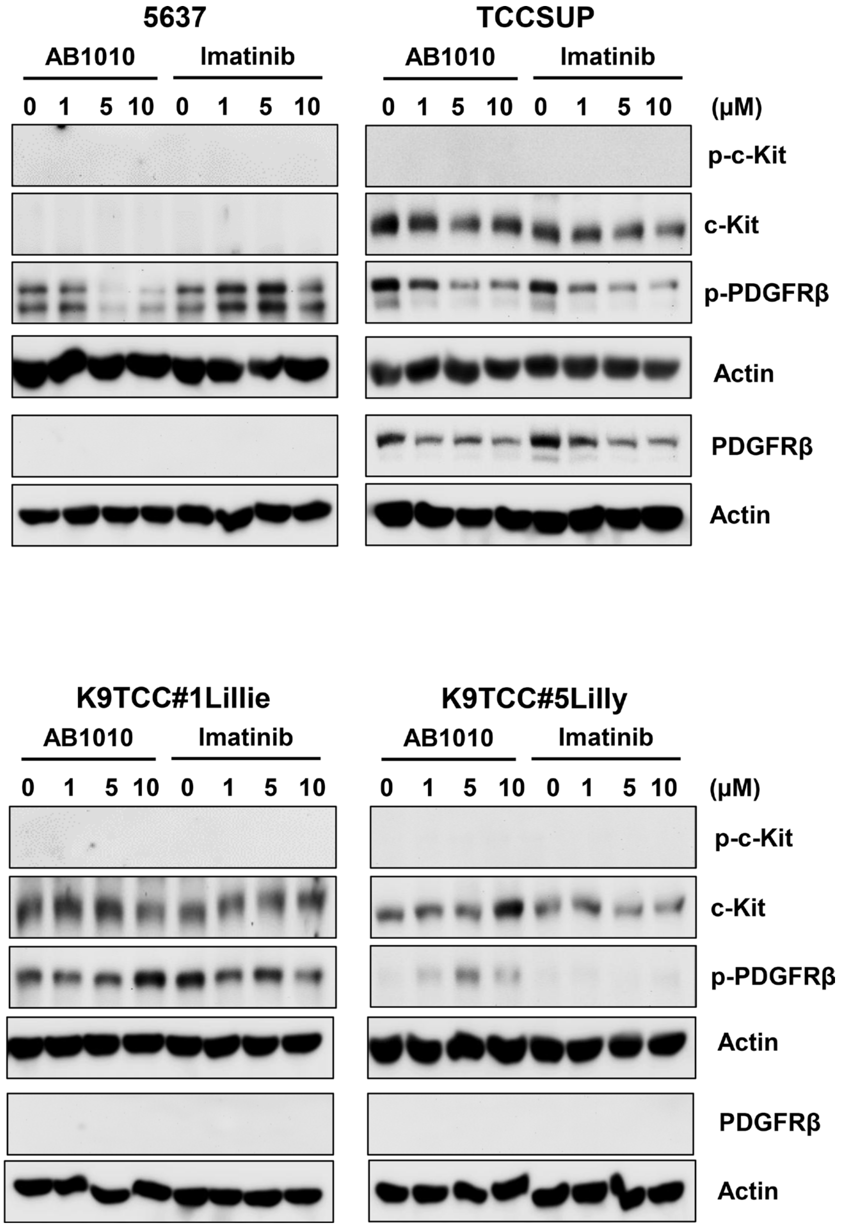

Supplementary Figure 1: AB1010 and imatinib inhibited c-Kit and/or PDGFRβ in tested COX-2-expressing bladder cancer cells in a dose-dependent manner. Human bladder cancer 5637 and TCCSUP cells and canine bladder cancer K9TCC#1Lillie and K9TCC#5Lilly cells were treated with 1, 5, and 10 μM dose of AB1010 or imatinib for 24 h. The expression of p-c-Kit, c-Kit, p-PDGFRβ, and PDGFRβ proteins were determined by WB analysis (*n* = 2). Actin was used as a loading control.
